# Robust Current Sensing in Rectangular Conductors: Elliptical Hall-Effect Sensor Array Optimized via Bio-Inspired GWO-BP Neural Network

**DOI:** 10.3390/s25103116

**Published:** 2025-05-15

**Authors:** Yue Tang, Jiajia Lu, Yue Shen

**Affiliations:** 1 School of Internet of Things Engineering, Wuxi University, Wuxi 214105, China; tangyue@cwxu.edu.cn; 2 School of Automation, Nanjing University of Information Science and Technology, Nanjing 210044, China; shenyue0900@163.com

**Keywords:** current sensing, mechanical deformation, error compensation, bio-inspired algorithm, grey wolf optimizer (GWO), Hall sensor array, 3D magnetic field reconstruction, electric vehicle powertrains

## Abstract

**Highlights:**

**What are the main findings?**

- A novel bio-inspired strategy: the integration of an elliptical Hall sensor array with a hybrid GWO-enhanced neural network achieves a robust suppression of mechanical deformation-induced errors (65.07%, 45.74%, and 76.15% for *X*/*Y*/*Z* deviations).
- A space-efficient design: the elliptical sensor configuration reduces the installation footprint by 72.4% compared to conventional circular arrays while maintaining a high accuracy under extreme deformations (±8 mm displacement, ±15° tilt).

**What are the implications of the main findings?**

- Critical for compact applications: enables precise current sensing in space-constrained systems, such as electric vehicle powertrains and miniaturized inverters, addressing the challenges of high-density power electronics.
- Algorithm–hardware synergy: bridges bio-inspired optimization and adaptive sensing hardware, offering a systematic framework for mitigating mechanical interference in real-world industrial settings.

**Abstract:**

Accurate current sensing in rectangular conductors is challenged by mechanical deformations, including eccentricity (X/Y-axis shifts) and inclination (Z-axis tilt), which distort magnetic field distributions and induce measurement errors. To address this, we propose a bio-inspired error compensation strategy integrating an elliptically configured Hall sensor array with a hybrid Grey Wolf Optimizer (GWO)-enhanced backpropagation neural network. The eccentric displacement and tilt angle of the conductor are quantified via a three-dimensional magnetic field reconstruction and current inversion modeling. A dual-stage optimization framework is implemented: first, establishing a BP neural network for real-time conductor state estimations, and second, leveraging the GWO’s swarm intelligence to refine network weights and thresholds, thereby avoiding local optima and enhancing the robustness against asymmetric field patterns. The experimental validation under extreme mechanical deformations (X/Y-eccentricity: ±8 mm; Z-tilt: ±15°) demonstrates the strategy’s efficacy, achieving a 65.07%, 45.74%, and 76.15% error suppression for X-, Y-, and Z-axis deviations. The elliptical configuration reduces the installation footprint by 72.4% compared with conventional circular sensor arrays while maintaining a robust suppression of eccentricity- and tilt-induced errors, proving critical for space-constrained applications, such as electric vehicle powertrains and miniaturized industrial inverters. This work bridges bio-inspired algorithms and adaptive sensing hardware, offering a systematic solution to mechanical deformation-induced errors in high-density power systems.

## 1. Introduction

The existing research on current sensing in electrical conductors predominantly focuses on circular arrays for cylindrical conductors, with studies [1,2,3,4,5,6,7,8] addressing eccentricity, tilt, and external interference via sensor redundancy and weighted compensation. However, rectangular conductors—favored in industrial applications for their superior thermal performance and cost efficiency—require disproportionately large circular arrays for accurate measurement, limiting their suitability in space-constrained scenarios [9]. Rectangular array configurations [10,11] and surface-mounted sensor designs [12,13,14] partially mitigate spatial constraints but introduce new challenges, including abrupt magnetic gradient shifts at corners and a sensitivity to positional errors. Regarding rectangular busbars, ref. [15] proposed a method for configuring sensor arrays through the rational selection of magnetic field detection points. For current measurement in diverse flat conductors, elliptic sensor arrays demonstrate significant space-saving advantages over circular counterparts [16]. Florian Zapf further contributed a simplified design of elliptic arrays with optimized crosstalk suppression [9]. In summary, elliptical arrays emerge as an optimal compromise, offering (1) 72.4% smaller footprints than circular counterparts, (2) gentler magnetic field gradients compared with rectangular layouts, and (3) an enhanced tolerance to mechanical deformations (eccentricity and tilt) via geometrically adaptive sampling. Despite the many advantages of elliptical arrays, the existing work generally assumes that the conductors are ideally placed vertically, ignoring the eccentricity and tilting errors caused by the mechanical vibration in practical applications, which limits their adaptability in large-operating-current (500A) measurements, such as high-voltage switchboards in electric vehicles [17].

Focusing on the above problem and the measurement of large currents (500A) in space-constrained environments, this study proposes a biomimetic error compensation strategy oriented to elliptical Hall-effect sensor arrays designed to measure the operating currents of rectangular conductors in confined spaces, such as high-voltage switchboards in electric vehicles. Our method integrates two key innovations: (1) 3D magnetic field modeling for the quantitative characterization of eccentricity and tilt parameters, and (2) a Grey Wolf Optimizer (GWO)-enhanced BP neural network for adaptive error suppression [18,19], overcoming the local optimal trap in traditional neural training. Based on the strategy for the better measurement of elliptical arrays in reference [9], geometric structure optimization, aspect ratio (AR) balance, and sensor density measurements are carried out on elliptical arrays to minimize the space occupied and maximize accuracy. This work establishes a framework for precise current sensing in the next generation of compact power systems by combining biomimetic algorithms with sensor array topology innovation.

## 2. Design of Elliptical Hall-Effect Sensor Arrays

### 2.1. Elliptical Array Configuration

In narrow-space current measurement scenarios for rectangular conductors, such as those in electric vehicle (EV) high-voltage distribution boxes, elliptical Hall-effect sensor arrays could be configured to support battery fault diagnosis and safety management. While standardized dimensions for these conductors remain undefined, the prevailing industrial practice adopts a classical rectangular cross-section of 20 mm × 4 mm. Given the spatial constraints of the circuit layers and compatibility requirements for diverse conductor sizes, the major semi-axis (a) of the elliptical array is empirically set to 30 mm. Since this paper’s research content is oriented toward engineering applications, this paper uses the AR for the ellipse definition to make it more intuitive and easier to understand, more convenient for geometric modeling and simulation, more suitable for parametric analysis, and more convenient for the modeling and control of actual structures or sensors. The AR is defined as the ratio of the minor semi-axis (b) to a (AR=b/a), which spans (0, 1), with circular arrays corresponding to an AR = 1.

Multiple Hall elements are distributed along a closed-loop path surrounding the current-carrying conductor. Their collective Hall voltage outputs reconstruct the magnetic field integral along this path, where the reconstruction fidelity depends on both the number and spatial arrangement of the sensors. This work employs the uniform curve segment length (UCSL) method to optimize the element placement, ensuring equidistant spacing along the elliptical contour. The UCSL method achieves balanced spatial sampling by maintaining uniform arc lengths between adjacent sensors, thereby minimizing positional asymmetry-induced errors.

Given the absence of an analytical solution for the arc length integration along elliptical closed-loop paths, this work adopts a numerical approximation approach. The elliptical contour is discretized into K densely sampled points ($K\geq 5\times 10^{6}$) [17]. Initialized from the first point nearest to the X-axis in the first quadrant, the coordinate of the n-th point is given using the following equation:

(1) $$E_{n}=(E_{n,x},E_{n,y})=(a\cos\frac{n\times 360^{\circ }}{K},b\sin\frac{n\times 360^{\circ }}{K}),n=1,2,\cdots ,K$$

The length from the X-axis to the *k*-th point is given via the following equation:

(2) $$s(k)=\sum_{n=1}^{k}\sqrt{{\left(E_{n,x}-E_{n-1,x}\right)}^{2}+{\left(E_{n,y}-E_{n-1,y}\right)}^{2}},k=1,2\cdots ,K$$

According to Equation (2), the circumference of an elliptical closed path is s(K), if the UCSL method is used to divide the elliptical closed path into *N* segments, the arc length of each segment is given by

(3) $$\Delta s=\frac{s(K)}{N}$$

The relative bias parameter of the curve segment is $s_{0,r}$, according to reference [14], and the optimal value calculation formula is

(4) $$s_{0,r}≅\left\{\begin{array}{c} \frac{1}{2\times 4N},N\;is\;an\;odd\;number\\ \;\;\frac{1}{4N}\;\;,\;\;N\;is\;an\;even\;number \end{array}\right.$$

The initial bias curve segment from the *X*-axis to the first Hall element has an arc length of $s_{0}$, which is given by

(5) $$s_{0}=s_{0,r}\times s(K)$$

The length of the curve segment from the *X*-axis to the *i*-th (i=1,2⋯,N) Hall element $P_{i-1}$ is given by

(6) $$s_{i}=s_{0}+i\Delta s$$

Given the arc length equivalence condition $s(m)=s_{i}$, m=1,2,⋯,K, where s(m) denotes the cumulative arc length from the *X*-axis to the *m*-th discretized point on the elliptical path and $s_{i}$ represents the predefined arc length to the *i*-th Hall element $P_{i-1}$, the coordinates of the Hall element $P_{i-1}$ are calculated as follows:

(7) $$P_{i-1}=(a\cos\frac{m\times 360^{\circ }}{K},b\sin\frac{m\times 360^{\circ }}{K}),n=1,2,\cdots ,K$$

The tangential vector of the elliptical curve at the Hall element $\vec{t_{i-1}}$, which is also the sensitive axis direction of the Hall element, can be given using the following equation:

(8) $$\vec{t_{i-1}}=\frac{\left(\begin{array}{c} -a\sin\frac{m\;\times \;360^{\circ }}{K}\\ b\cos\frac{m\;\times \;360^{\circ }}{K} \end{array}\right)}{\sqrt{{\left(a\sin\frac{m\;\times \;360^{\circ }}{K}\right)}^{2}+{\left(b\cos\frac{m\;\times \;360^{\circ }}{K}\right)}^{2}}}$$

**Figure 1:**
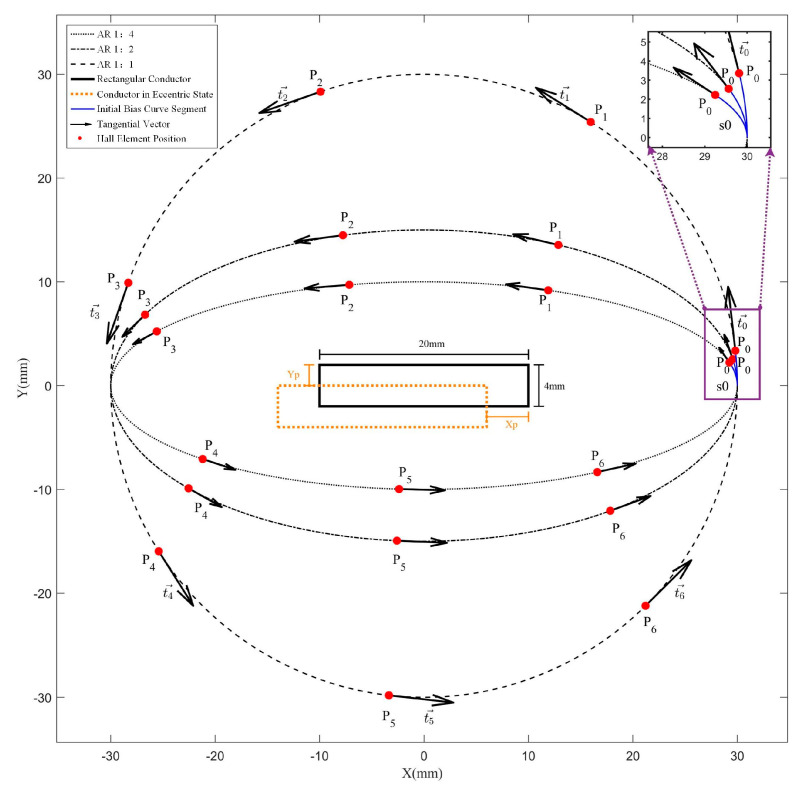
Schematic diagram of Hall-effect sensor arrays with different AR.

Figure 1 illustrates comparative schematic diagrams of elliptical Hall-effect sensor arrays with varying ARs, where $s_{0}$ denotes the initial offset curve segment, $P_{i-1}$ denotes the position of the Hall element, $\vec{t_{i-1}}$ denotes the sensitive axis direction of the Hall element, and $X_{p}$ and $Y_{p}$ denote the eccentricity displacements of the conductor along the *X*-axis and *Y*-axis directions, respectively.

### 2.2. Theoretical Calculation

#### 2.2.1. Operating Principle of Magnetic Sensor Array-Based Current Sensing

The magnetic field generated by a long straight current-carrying conductor can be treated as a quasi-static field, satisfying Ampère’s Circuital Law:

(9) ∮Hdl=∑I

where *H* denotes the magnetic field intensity and *I* represents the measured current.

By deploying *N* Hall sensors along the integration path using the UCSL method, the measured current can be derived from

(10) $$I=\sum_{i=1}^{N}H_{i}\Delta s_{i}$$

$H_{i}$ is the tangential magnetic field intensity at the *i*-th Hall element, and $\Delta s_{i}$ corresponds to the arc length of each curve segment. For the UCSL distribution, all segments maintain equal arc lengths ($\Delta s_{i}$ = constant), enabling a direct current computation through a magnetic field distribution analysis along the conductor.

#### 2.2.2. Current Measurement Principle for Rectangular Conductors

**Figure 2:**
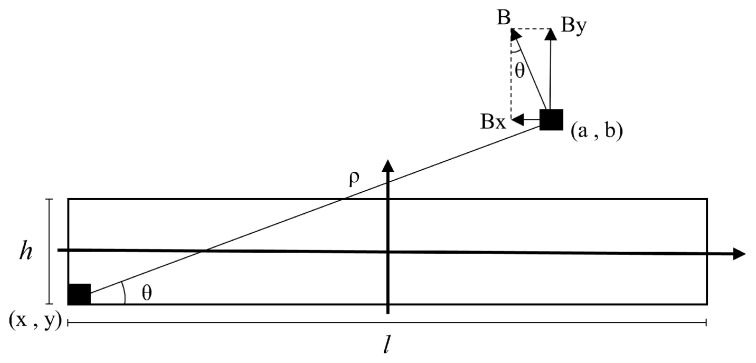
The magnetic field of a rectangular cross-section conductor excited at any point of the plane.

As illustrated in Figure 2, a Cartesian coordinate system is established with the centroid of the rectangular conductor as the origin. Owing to the geometric characteristics of both the rectangular conductor and the elliptical array, the magnetic field distribution around the current-carrying rectangular conductor exhibits symmetry only with respect to the *X*- and *Y*-axes. Consequently, each Hall element distributed along the elliptical closed-loop path senses distinct tangential magnetic field components, resulting in non-uniform Hall voltage outputs.

However, since the current distribution across the conductor’s cross-section is uniform (i.e., constant current density), the magnetic field at any planar point (*a*, *b*) can be derived via the infinitesimal element method. Specifically, the total magnetic field intensity generated by the rectangular conductor is calculated as the vector superposition of contributions from all differential current elements.

Based on the infinitely long straight current-carrying conductor model, the magnetic field intensity *H* at a radial distance *ρ* from the conductor axis is given by

(11) $$H_{i}=\frac{I}{2\pi \rho }$$

For a rectangular current-carrying conductor with a cross-sectional area *S*, the differential current element is defined as follows:

(12) $$dI=\frac{I}{S}dxdy$$

The *X*-axis component of the magnetic field intensity generated by *dI* at point (*a*, *b*) is

(13) $$dH_{ix}=-\frac{dI}{2\pi \rho }\sin\theta =-\frac{I\sin\theta }{2\pi \rho S}dxdy$$

Integrating over the conductor’s cross-section, the total *X*-axis magnetic field component at (*a*, *b*) becomes

(14) $$\begin{array}{l} H_{ix}=-\iint_{S}\frac{I\sin\theta }{2\pi \rho S}dxdy\\ \;=-\iint_{S}\frac{I(b-y)}{2\pi S[{\left(a-x\right)}^{2}+{\left(b-y\right)}^{2}]}dxdy\\ \;=-\frac{I}{2\pi S}\int_{-h/2}^{h/2}dy\int_{-l/2}^{l/2}\frac{\left(b-y\right)}{{\left(a-x\right)}^{2}+{\left(b-y\right)}^{2}}dx \end{array}$$

Similarly, the *Y*-axis component is

(15) $$\begin{array}{l} H_{iy}=\iint_{S}\frac{I\cos\theta }{2\pi \rho S}dxdy\\ \;=\iint_{S}\frac{I(a-x)}{2\pi S[{\left(a-x\right)}^{2}+{\left(b-y\right)}^{2}]}dxdy\\ \;=\frac{I}{2\pi S}\int_{-h/2}^{h/2}dy\int_{-l/2}^{l/2}\frac{\left(a-x\right)}{{\left(a-x\right)}^{2}+{\left(b-y\right)}^{2}}dx \end{array}$$

The resultant magnetic field intensity vector at (*a*, *b*) is

(16) $$\vec{H_{i}}=\left(\begin{array}{c} H_{ix}\\ H_{iy} \end{array}\right)$$

The magnetic field component along the Hall element’s sensitive axis direction is

(17) $$H_{i,MFS}=\vec{H_{i}}\cdot \vec{t_{i}}$$

Applying Ampère’s Circuital Law, the calculated current is obtained by

(18) $$I_{calc}=\sum_{i=1}^{N}H_{i,MFS}\cdot \Delta s_{i}$$

The relative current measurement error is defined as

(19) $$\varepsilon_{calc}=(\frac{I_{calc}-I}{I})\times 100\%$$

**Figure 3:**
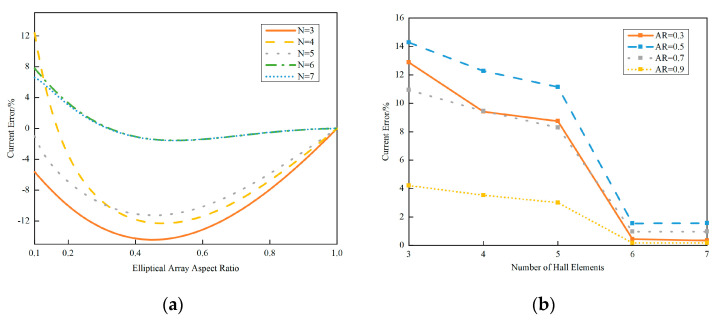
Plot of elliptical array aspect ratio versus current error. (**a**) Elliptical array aspect ratio and current error and (**b**) Hall element count and current error.

MATLAB (R2020a)-based parametric sweeps were performed to identify the optimal AR and Hall element count (N) for space-constrained applications. The calculated relationship between the AR and $\varepsilon_{calc}$ is shown in Figure 3.

As revealed in Figure 3a, the theoretical current error reduces to zero when the elliptical array adopts an aspect ratio (AR) of 1:1 (i.e., circular configuration). However, circular arrays demand excessive spatial footprints, rendering them impractical for confined installations. Notably, alternative AR values achieving zero errors exist for specific sensor counts: N = 4 with an AR = 0.17; N = 6 with an AR = 0.33; and N = 7 with an AR = 0.31.

The N = 4 and AR = 0.17 combination is excluded due to limited its adaptability to rectangular conductors of varying dimensions.

Figure 3b demonstrates that an increasing sensor density (N) improves the reconstruction fidelity to Ampère’s Circuital Law, thereby reducing current errors. However, diminishing returns emerge between N = 6 and N = 7, suggesting marginal improvements for N > 7. Considering cost-effectiveness and spatial constraints, this study focuses on the N = 6 and N = 7 configurations for subsequent optimization.

### 2.3. Simulation Setup

The accurate analysis of magnetic field distributions in rectangular current-carrying conductors—critical for current measurement precision—is more complex compared with circular conductors. Finite element simulations were performed to validate the theoretical models using COMSOL Multiphysics^®^ 6.1 on a Lenovo Y7000P laptop (CPU: Intel i7-12700H; GPU: NVIDIA RTX 3050, Lenovo, Hefei, China). Key simulation parameters included the following:

- Conductor Properties: rectangular copper busbar with an electrical conductivity of σ = 5.998 × 10^7^ S/m and a density of ρ = 8960 kg/m^3^;
- Ambient Medium: non-conductive air (σ = 0 S/m);
- Probe Placement: Hall element positions equipped with magnetic flux density probes;
- Mesh Strategy: adaptive mesh refinement prioritizing boundary layers near the conductor edges.

**Figure 4:**
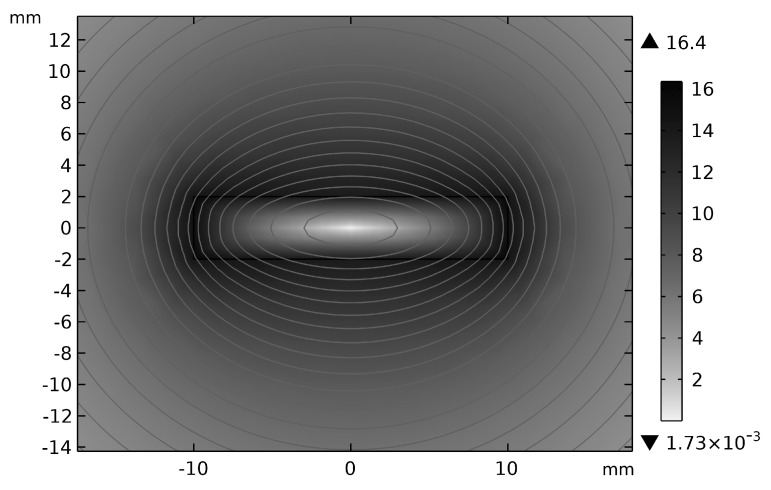
Magnetic field distribution of rectangular current-carrying conductor.

The simulated magnetic flux density modulus distribution (Figure 4) exhibits quasi-elliptical contours around the conductor, corroborating the feasibility of elliptical Hall array configurations. This spatial alignment between the field geometry and sensor topology minimizes reconstruction errors in Ampère’s integral approximations.

**Figure 5:**
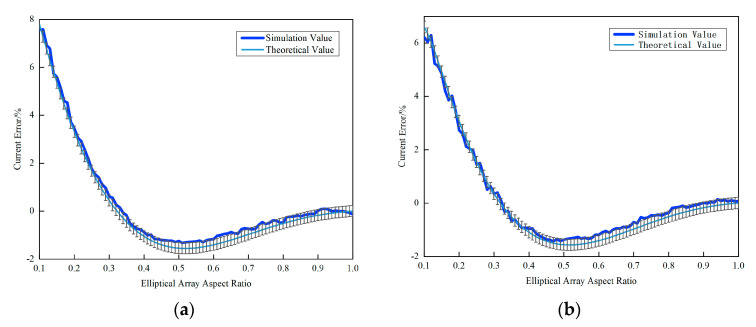
Elliptical array aspect ratio and current error simulation results. (**a**) When N = 6, AR = 0.33 and (**b**) When N = 7, AR = 0.31.

The COMSOL Multiphysics^®^-MATLAB LiveLink™ interface was leveraged to establish a bidirectional co-simulation framework. Figure 5 demonstrates the simulated relationship between the aspect ratio (AR) and current error for elliptical arrays with N = 6 and N = 7 sensor configurations. Despite inherent simulation artifacts arising from mesh discretization methods and numerical convergence thresholds, the results exhibit a strong agreement with theoretical predictions—all error values reside within ±0.18% of the theoretical standard error bounds.

## 3. GWO-BP-Based Error Optimization Algorithm

### 3.1. Parameter Initialization

**Figure 6:**
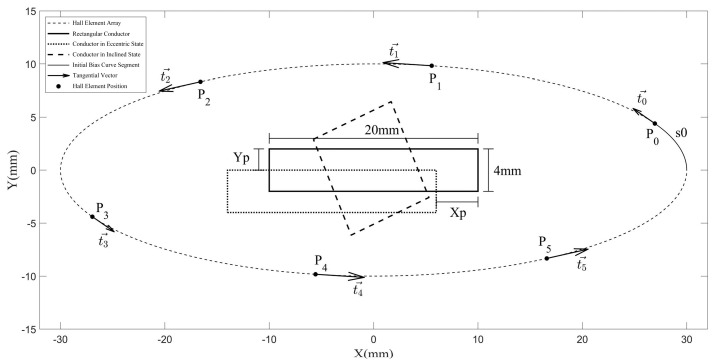
A schematic diagram of a 6-element elliptical Hall-effect sensor array.

The elliptical Hall-effect sensor array is geometrically defined with a major semi-axis, a = 30 mm, and minor semi-axis, b = 10 mm. Six Hall elements are distributed around the rectangular conductor using the UCSL method. Their position $P_{i-1}$ (i=1,2⋯,6) and the angular offset $\omega_{i}$ is between the sensitive axis and the *X*-axis (see Table 1 for numerical values). Figure 6 illustrates the array configuration, where s0 is the baseline offset curve segment for the zero-current calibration; $P_{i-1}$ denotes the position of the Hall element, $\vec{t_{i-1}}$ denotes the sensitive axis direction of the Hall element; and $X_{p}$ and $Y_{p}$ denote the eccentricity displacements of the conductor along the *X*-axis and *Y*-axis directions, respectively.

### 3.2. Current Inversion Model

**Figure 7:**
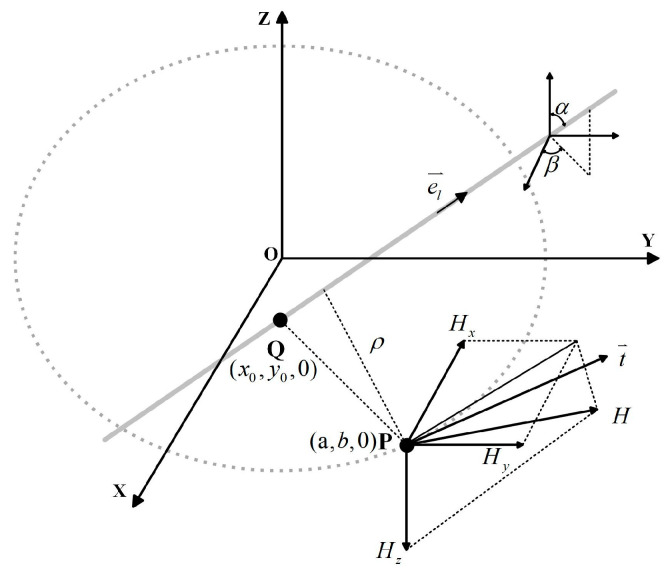
Current measurement schematic.

The schematic of the three-dimensional magnetic field-based current measurement is depicted in Figure 7. Q$\left(x_{0},y_{0},0\right)$ is the intersection point between the central axis of the conductor and the XY-plane, that is, $x_{0}$ represents the abscissa of the intersection point between the central axis of the conductor and the XY-plane, and $y_{0}$ represents the ordinate of the intersection point between the central axis of the conductor and the XY-plane. In the experiment, $X_{p}$ and $Y_{p}$ are random, and $x_{0}$ and $y_{0}$ change with the change in the X-direction eccentricity $X_{p}$ and Y-direction eccentricity $Y_{p}$. α is the angle between the conductor’s central axis and the positive *Z*-axis (tilt deviation); β is the angle between the projection of the central axis onto the *XY*-plane and the positive *X*-axis (azimuthal misalignment).

This parametric framework enables a precise geometric decomposition of conductor misalignments, which is critical for reconstructing current distributions under arbitrary spatial offsets.

Incorporating spatial offsets caused by the conductor eccentricity and tilt, Equation (11) is modified to

(20) $$H_{i}=\frac{I}{2\pi \rho }\vec{e_{l}}\times \vec{e_{r}}$$

where $\vec{e_{r}}$ is the unit directional vector of the relative position between (a, b, 0) and the conductor, $\vec{e_{l}}$ is the direction of the conductor, and $\vec{e_{l}}=(m,n,p)$ where m=sin(α)cos(β), n=sin(α)sin(β), and p=cos(α).

When $\vec{QP}$ denotes the vector from point Q to the Hall element located at P (a,b,0), the unit direction vector of the magnetic field intensity $H_{i}$ is then expressed as

(21) $$\vec{e_{\varphi }}=\vec{e_{l}}\times \vec{e_{r}}=\frac{\rho }{\left|\rho \right|}=\frac{\vec{e_{l}}\times \vec{QP}}{\left|\vec{e_{l}}\times \vec{QP}\right|}$$

The *X*-axis component of the magnetic field intensity generated by the measured current *I* at point (a, b, 0) is expressed as

(22) $$H_{ix}=\frac{I}{2\pi A}\iint_{S}\frac{-p(b-y)}{p^{2}{\left(a-x\right)}^{2}+p^{2}{\left(b-y\right)}^{2}+{\left[m(a-x)-n(b-y)\right]}^{2}}dxdy$$

**Figure 8:**
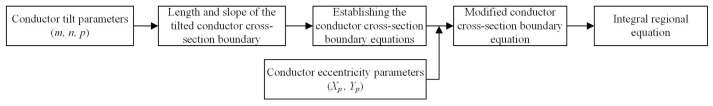
A flowchart for calculating the integral region equations.

The computational workflow for determining the integration domain S is illustrated in Figure 8. First, cross-sectional boundary equations are constructed from tilt parameters (α,β), which are then adjusted for eccentricity offsets $\left(X_{p},Y_{p}\right)$. This iterative process yields the generalized cross-sectional equation of the conductor under arbitrary positional states, ultimately defining the integration domain equation for magnetic field computations.

The component of the magnetic field strength on the *Y*-axis is

(23) $$H_{iy}=\frac{I}{2\pi A}\iint_{S}\frac{p(a-x)}{p^{2}{\left(a-x\right)}^{2}+p^{2}{\left(b-y\right)}^{2}+{\left[m(a-x)-n(b-y)\right]}^{2}}dxdy$$

The component of the magnetic field strength on the *Z*-axis is

(24) $$H_{iz}=\frac{I}{2\pi A}\iint_{S}\frac{m(a-x)-n(b-y)}{p^{2}{\left(a-x\right)}^{2}+p^{2}{\left(b-y\right)}^{2}+{\left[m(a-x)-n(b-y)\right]}^{2}}dxdy$$

The magnetic field intensity components of the current I to be measured on the sensitive axes in the X-, Y-, and Z-directions at the Hall element $P_{i-1}$ (i=1,2⋯,6) are expressed as

(25) $$H_{ix,MFS}=H_{ix}\cos\omega_{i}$$

(26) $$H_{iy,MFS}=H_{iy}\sin\omega_{i}$$

(27) $$H_{iz,MFS}=H_{iz}$$

The magnetic field strength output by the Hall element $P_{i-1}$ (i=1,2⋯,6) is expressed as

(28) $$H_{i}=\sqrt{{H_{ix}}^{2}+{H_{iy}}^{2}+{H_{iz}}^{2}}$$

According to Ampere’s Loop Theorem, the calculated value of the current to be measured can be obtained as follows:

(29) $$I_{calc}=\sum_{i=0}^{5}H_{i}\cdot \Delta s$$

where Δs=22.13.

### 3.3. Estimation of Conductor Eccentricity and Tilt Parameters

The backpropagation (BP) neural network employs gradient descent for weight and threshold adjustments [18], demonstrating efficacy in nonlinear regression and classification tasks. However, the suboptimal initialization of weights and thresholds often traps the network in local optima, compromising the convergence reliability. To address this limitation, this work introduces a metaheuristic algorithm to optimize the initial weights and thresholds of the BP network, thereby enhancing its regression capability for multi-parameter estimations under mechanical deformations.

#### 3.3.1. Grey Wolf Optimizer (GWO)

The Grey Wolf Optimizer (GWO) is a metaheuristic search algorithm inspired by the hierarchical hunting behavior of grey wolf packs [19]. In the GWO, individuals are classified into four hierarchical roles (α, β, δ, and ω) based on their fitness values. Let $X_{p}(t)$ denote the position vector of the prey (optimal solution) and X(t) represent the position vector of the ω wolf at the *t*-th iteration. The distance *D* between the prey and the ω wolf is defined as

(30) $$\left\{\begin{array}{l} D=\left|C\cdot X_{p}(t)-X(t)\right|\\ C=2r_{2} \end{array}\right.$$

(31) $$\left\{\begin{array}{l} X(t+1)=X_{p}(t)-A\cdot D\\ A=2ar_{1}-a\\ a=\frac{2(T-t)}{t} \end{array}\right.$$

In the equations, A and C are collaborative coefficient vectors governing the swarm coordination, $r_{1}$ and $r_{2}$ are random numbers within the interval [0, 1], and T denotes the maximum number of iterations.

The grey wolves dynamically adjust their positions to encircle the prey (optimal solution), with the positions of the α, β, and δ wolves guiding the ω wolves’ movement. Let $D_{i-\omega },(i=\alpha ,\beta ,\delta )$ denote the distance between the *i*-th wolf and the ω wolf, $X_{i}(t),(i=\alpha ,\beta ,\delta )$ denote the position vector of the *i*-th wolf at the t-th iteration, $X_{i-\omega },(i=\alpha ,\beta ,\delta )$ denote the distance between the *i*-th wolf and the ω wolf at the *t*-th iteration, and X(t+1) denote the position vector of the ω wolf for the next iteration. The position update equations for the grey wolves are formulated as follows:

(32) $$\left\{\begin{array}{l} D_{\alpha -\omega }=\left|C_{1}\cdot X_{\alpha -\omega }(t)-X(t)\right|\\ D_{\beta -\omega }=\left|C_{2}\cdot X_{\beta -\omega }(t)-X(t)\right|\\ D_{\delta -\omega }=\left|C_{3}\cdot X_{\delta -\omega }(t)-X(t)\right| \end{array}\right.$$

(33) $$\left\{\begin{array}{l} X_{\alpha }=X_{\alpha -\omega }(t)-A_{1}\cdot D_{\alpha -\omega }\\ X_{\beta }=X_{\beta -\omega }(t)-A_{2}\cdot D_{\beta -\omega }\\ X_{\delta }=X_{\delta -\omega }(t)-A_{3}\cdot D_{\delta -\omega } \end{array}\right.$$

(34) $$X(t+1)=\frac{1}{3}(X_{\alpha }+X_{\beta }+X_{\delta })$$

The Grey Wolf Optimizer (GWO) employs stochastic collaborative coefficient vectors A to expand the search range of wolves during prey exploration, thereby enabling global search capabilities. Simultaneously, it incorporates a random vector C with values uniformly sampled from the interval [0, 2], which either amplifies or dampens the positional influence of the prey (optimal solution). This dual mechanism effectively prevents the iterative process from converging to local optima, ensuring a robust exploration–exploitation balance throughout the optimization.

#### 3.3.2. GWO-BP Conductor State Parameter Estimation Model

**Figure 9:**
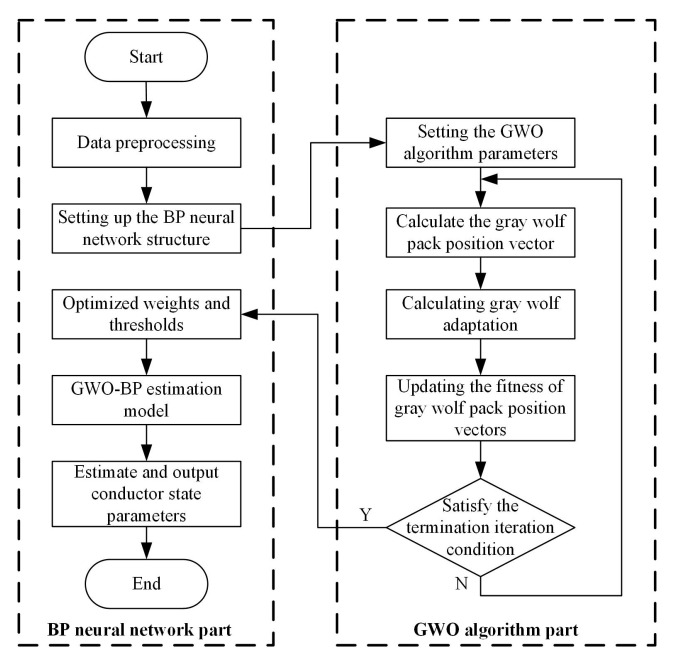
Flowchart of conductor state parameter estimation algorithm based on GWO-BP.

This study integrates the Grey Wolf Optimizer (GWO) with a backpropagation (BP) neural network to establish a robust model for estimating conductor state parameters under mechanical deformations, with the hybrid algorithm workflow illustrated in Figure 9 comprising dataset partitioning into training/testing sets followed by normalization; the determination of input/output layer nodes (12/5) based on magnetic field and conductor state parameters, respectively, and hidden layer nodes (11) via a trial-and-error optimization to construct a 12-11-5 BP neural network; the configuration of GWO parameters (population size = 20 and max iterations = 50) where the BP network weights/thresholds are encoded as prey position vectors for iterative wolf pack updates; and the final assignment of optimized parameters to build the GWO-BP hybrid model.

## 4. Experimental Design and Validation

### 4.1. Experimental Platform

**Figure 10:**
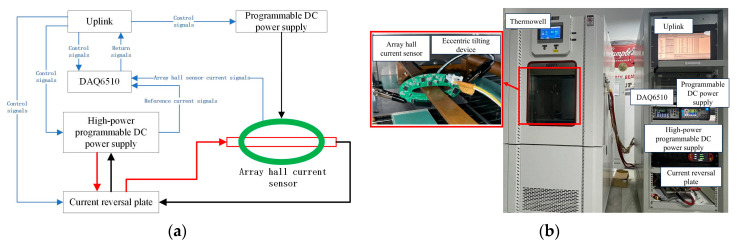
The experimental platform. (**a**) A schematic diagram of the experimental setup. (**b**) A physical drawing of the experimental setup.

The experimental platform, designed and validated within the LabVIEW environment for signal transmission and system integration [20], incorporates a Keithley DAQ6510 data acquisition system with 80-channel capacity, 16-bit resolution, and 1 MS/s sampling rate (16-bit ADC) for multi-channel data transfer to the host computer, an AMETEK SG Series modular DC power supply configured with six modules to deliver 500 A DC current (extendable to ±500 A via a current commutator board) to the rectangular conductor across a voltage range of 10–1000 V and current range of 5–6000 A (total power output: 30 kW) and programmable DC power supplies (0–24 V/0–5 A) for Hall-effect sensor array excitation, with system control signals generated by the host computer and experimental schematics/implementation shown in Figure 10.

### 4.2. Performance Evaluation of Conductor State Estimation Models

The performance of the BP neural network and GWO-BP algorithms was evaluated using three metrics: Mean Absolute Error (MAE), Mean Squared Error (MSE), and Mean Absolute Percentage Error (MAPE). Experimental results are summarized in Table 2.

Experimental results demonstrate that although the MAPE of the $X_{p}$ estimates from the proposed GWO-BP algorithm are marginally higher than that of the BP algorithm, the GWO-BP exhibits superior performance in MAE, MSE, and MAPE metrics for $Y_{p}$, m, n, and p estimations, thereby achieving enhanced precision in conductor state parameter estimation.

### 4.3. Experimental Results and Analysis

The rectangular conductor was positioned at the center of the array for a standard current and initial position calibration (no eccentricity or tilt) before each test. Subsequently, eccentricity and tilt were introduced for algorithm validation testing. Current pulses were configured with a 500 ms duration and 1s pause intervals to mitigate the thermal interference from the prolonged energization. The total output voltage was linearly fitted against the current via a least squares regression to extract the Hall array’s sensitivity and offset parameters. Subsequently, a programmable eccentricity–tilt adjustment mechanism was employed to induce mechanical deformations (X/Y-axis eccentricity: ±8 mm; Z-axis tilt: ±15°). Current measurements were performed under these perturbations, with measurement errors optimized independently using the BP and GWO-BP algorithms.

**Figure 11:**
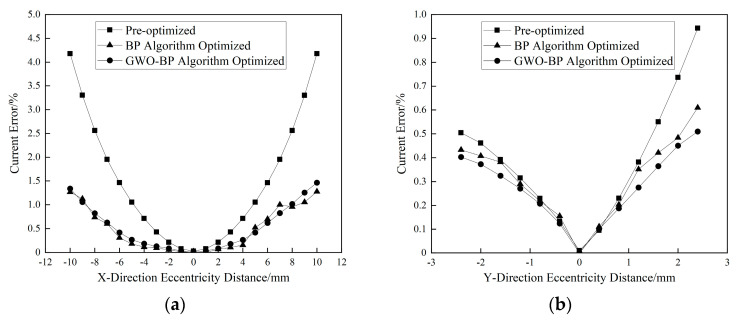
Conductor eccentricity distance vs. error before and after optimization. (**a**) Plot of *X*- Dir. eccentricity distance vs. error; (**b**) Plot of *Y*- Dir. eccentricity distance vs. error.

Figure 11a illustrates the relationship between the *X*-axis eccentricity displacement and the current measurement error. The error escalates with increasing eccentricity, reaching 4.18% at 10 mm displacement without optimization. Post-optimization, the BP algorithm reduces this error to 2.83%, while the GWO-BP algorithm achieves a significantly lower error of 1.46%, corresponding to a 65.07% error suppression relative to the baseline. Figure 11b analyzes *Y*-axis eccentricity effects. For displacements ≤ 0.4 mm, both optimized and non-optimized errors remain comparable (Δ < 0.05%). Beyond this threshold, the GWO-BP algorithm demonstrates progressive superiority, reducing the error from 0.94% (unoptimized) to 0.51% at a 2.4 mm displacement—a 45.74% improvement.

**Figure 12:**
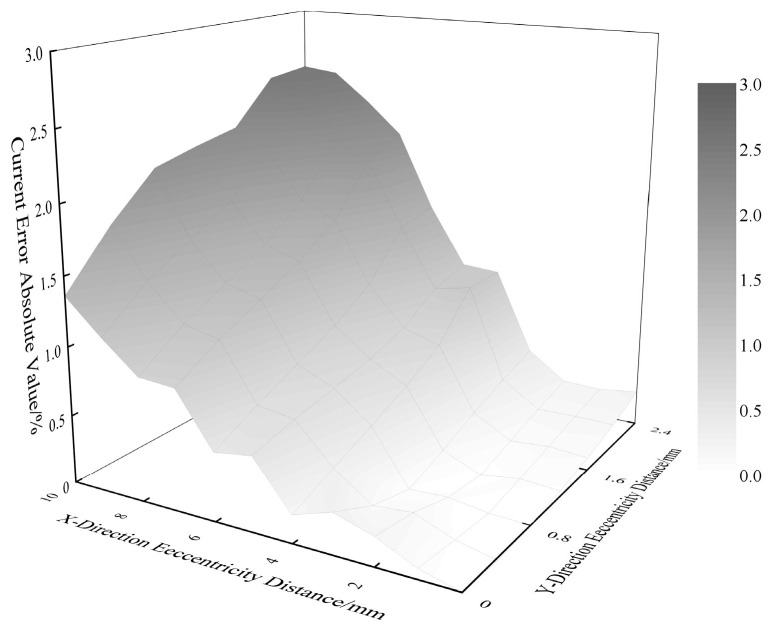
Conductor eccentricity distance versus error after error optimization by GWO-BP algorithm.

Figure 12 presents the GWO-BP-optimized error profile across the full permissible eccentricity range (X/Y: ±8 mm). The maximum current measurement error is constrained to 2.54%, validating the algorithm’s robustness under extreme mechanical deformations.

**Figure 13:**
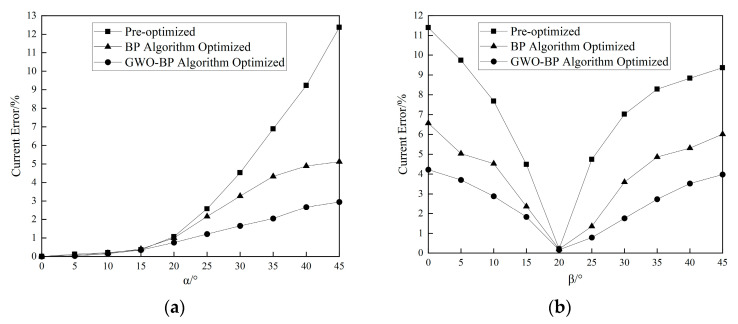
Conductor tilt angle vs. error before and after optimization. (**a**) Plot of α-angle vs. error. (**b**) Plot of β-angle vs. error.

Figure 13a demonstrates the α-angle versus the current error relationship at β = 20°. For α > 15°, the current error escalates rapidly with increasing α, indicating amplified measurement inaccuracies under severe *Z*-axis deviations. At α = 45°, the unoptimized error reaches 12.37%, while the GWO-BP algorithm limits it to 2.95%—a 76.15% error suppression. Figure 13b analyzes β-angle impacts at α = 0°. Since the magnetic induction intensity distribution around the conductor is more matched with the sensor layout direction when β is about 20°, the difference in sampling values between sensors is small, and the neural network fitting is more stable. Therefore, there is a local minimum value of the current error near β = 20°. The GWO-BP algorithm achieves a 62.92% local error reduction in this critical region, markedly improving the tilt-induced error resilience.

**Figure 14:**
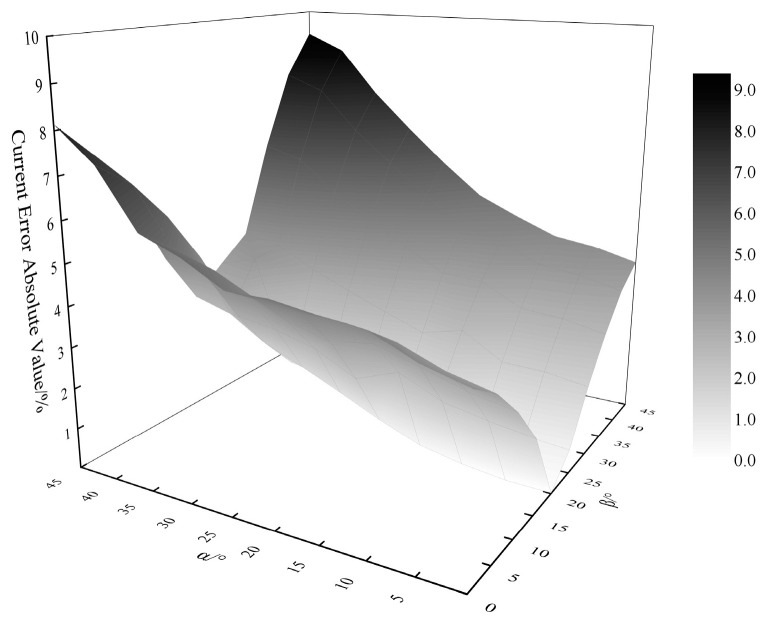
Conductor tilt angle versus error after optimization by GWO-BP algorithm.

Figure 14 presents the GWO-BP-optimized error profile across the full permissible tilt range (α: ±45°; β: ±30°), constraining the maximum current error to 9.37% under extreme angular misalignments.

The proposed eccentricity–tilt error optimization algorithm achieves a precise estimation of conductor positional parameters (X_p_, Y_p_, α, β), effectively reducing current measurement errors by 65.07% (X-eccentricity), 45.74% (Y-eccentricity), and 76.15% (Z-tilt), thereby advancing the elliptical Hall sensor array performance for high-density power systems.

From the results of conductor eccentricity and tilt experiments, it can be seen that the current sensor designed in this paper and its error optimization algorithm have a better error suppression effect for the *X*-axis and *Z*-axis direction, while the error suppression effect for the *Y*-axis direction is slightly worse, which is mainly due to the following reasons:

(1) The spatial layout of the sensor array: The elliptical array designed in this manuscript has an aspect ratio of 0.33, and the short-axis direction (i.e., Y-axis) is less spaced from the conductor, so that the sensor’s response to changes in the magnetic field in the *Y*-axis is more sensitive, and thus the current error in the *Y*-axis is inherently smaller before optimization. In contrast, the initial errors in the *X*-axis and *Z*-axis are larger, possessing more room for optimization, which results in a more significant error suppression in these two directions after optimization.

(2) The sensitivity of the GWO-BP model to different directional perturbations: Considering the small error suppression space of the *Y*-axis and the computational complexity of the algorithm, we focus on the optimization of the *X*-axis and *Z*-axis when designing the optimization algorithm. Therefore, when designing the GWO-BP model, the fitting ability of the main error components of the *X*-axis and *Z*-axis is preferentially enhanced, while the fitting weight of the small amplitude disturbance in the *Y*-axis direction is relatively low. This strategy affects the error suppression effect of the *Y*-axis to some extent.

The experimental results show that the current sensor designed in this paper and the GWO-BP compensation algorithm have achieved good results in current error suppression, but there is still room for further improvement. Based on the theoretical analysis and experimental results in this paper, we believe that subsequent optimization can be carried out in the following three aspects in the future:

(1) Algorithm level optimization: The current GWO-BP model tends to prioritize the suppression of errors in major directions (e.g., the X- and Z-axes) during the training process. A multi-objective optimization strategy or the construction of a weighted loss function can be introduced to achieve the balanced optimization of multi-direction errors and enhance the model’s adaptability in error suppression in secondary directions, such as the Y-axis.

(2) Sensor array design optimization: considering the non-uniformity of the magnetic field distribution in different directions, the aspect ratio parameter of the elliptical array can be further optimized or the non-uniform deployment scheme can be adopted in the subsequent stage, so as to enhance the system’s responsiveness and robustness to local perturbations.

(3) Data processing and model training: the current training set has a certain degree of uneven distributions of samples under some working conditions, and data enhancement techniques can be used to expand the sample dimension and distribution density, so as to improve the model’s generalization ability and adaptability to unknown working conditions.

In addition, due to factors such as the sensor installation error, experimental platform accuracy, and fitting model error, new error sources may be introduced during the experiment. Combined with the experimental process and system design of this paper, the main error sources can be summarized as follows:

(1) Sensor installation error: Position deviations and attitude errors will inevitably occur during the deployment process because the direction of the sensitive axis of the Hall sensor is angle-dependent, and multiple sensors need to be deployed at different positions of the elliptical array in the experiment. This results in the magnetic field sampling value being inconsistent with the theoretical expectation, which affects the input accuracy of the fitting model.

(2) Modeling deviation of the conductor’s actual offset and tilt error: In the experiment, we simulated different eccentricity and tilt states using a precision platform. However, the ideal modeling state and the actual test state slightly differed due to the limited resolution of the actual platform adjustment and the possible existence of small mechanical gaps in the physical structure. This difference would also be reflected in the generalization ability of the error compensation model.

(3) Fitting model error: although we use a GWO optimization strategy to improve the convergence performance and global search ability of the BP neural network, the training data distribution, weight initialization, and super parameter setting of the BP neural network may still lead to a certain degree of under-fitting or over-fitting, which will affect the stability and consistency of the error compensation.

## 5. Conclusions

This study develops an eccentricity and tilt error optimization strategy for elliptical Hall-effect current sensor arrays by integrating a bio-inspired GWO-enhanced BP neural network with three-dimensional magnetic field modeling and current inversion theory. The proposed framework establishes a conductor state estimation model through a swarm intelligence-driven optimization of neural network parameters, effectively addressing magnetic field distortions caused by mechanical deformations in rectangular conductors. The experimental validation confirms the superiority of the GWO-BP algorithm in achieving a robust error suppression across multi-axis deviations. The optimized elliptical array configuration significantly reduces the spatial occupancy compared to traditional circular or rectangular arrays while maintaining immunity to complex geometric misalignments. By synergizing an adaptive algorithm design with sensor array topology innovations, this work advances high-accuracy current sensing in geometrically constrained scenarios, particularly enhancing reliability for emerging applications, such as electric vehicle battery management systems.

## Figures and Tables

**Table 1:** Hall element positions.

	$\mathrm{Hall}\;\mathrm{Element}\;\mathrm{Position}\;P_{i-1}$	$\mathrm{Angle}\;\mathrm{of}\;\mathrm{Rotation}\;\mathrm{of}\;\mathrm{the}\;X\text{-}\mathrm{Direction}\;\mathrm{Sensitive}\;\mathrm{Axis}\;\omega_{i}$
$P_{0}$	(26.94, 4.40, 0)	145.79°
$P_{1}$	(5.57, 9.83, 0)	176.38°
$P_{2}$	(−16.59, 8.33, 0)	192.58°
$P_{3}$	(−26.94, −4.40, 0)	325.79°
$P_{4}$	(−5.56, −9.83, 0)	356.38°
$P_{5}$	(16.59, −8.33, 0)	12.58°

**Table 2:** Comparison of performance metrics for different algorithms.

Estimated Parameters	Estimation Algorithms	Evaluation Indicators		
		MAE	MSE	MAPE
$X_{p}$	BP	0.2272	0.0771	0.8419%
	GWO-BP	0.0498	0.0041	1.0411%
$Y_{p}$	BP	0.0619	0.0043	5.2646%
	GWO-BP	0.0146	0.0004	1.0621%
m	BP	0.0124	0.0003	10.3490%
	GWO-BP	0.0037	0.0001	2.8585%
n	BP	0.0071	0.0002	3.9567%
	GWO-BP	0.0028	0.0001	0.8626%
p	BP	0.0143	0.0005	2.2479%
	GWO-BP	0.0029	0.0001	0.4173%

## Data Availability

The data are not publicly available as they are required for further research. Requests for access could be directed to the corresponding author.

## Abbreviations

The following abbreviations are used in this manuscript:

GWO	Grey Wolf Optimizer
EV	electric vehicle
AR	aspect ratio
UCSL	uniform curve segment length
MAE	Mean Absolute Error
MSE	Mean Squared Error
MAPE	Mean Absolute Percentage Error
